# Improving Menstrual Health Literacy Through Life-Skills Programming in Rural Ethiopia

**DOI:** 10.3389/fgwh.2022.838961

**Published:** 2022-07-08

**Authors:** Sarah Baird, Joan Hamory, Kiya Gezahegne, Kate Pincock, Tassew Woldehanna, Workneh Yadete, Nicola Jones

**Affiliations:** ^1^Department of Global Health, George Washington University, Washington, DC, United States; ^2^Department of Economics, University of Oklahoma, Norman, OK, United States; ^3^Department of Social Anthropology, Addis Ababa University, Addis Ababa, Ethiopia; ^4^Gender and Adolescence: Global Evidence (GAGE), ODI, London, United Kingdom; ^5^Department of Economics, Addis Ababa University, Addis Ababa, Ethiopia; ^6^Gender and Adolescence: Global Evidence (GAGE), Quest Consulting, Addis Ababa, Ethiopia

**Keywords:** adolescence, Ethiopia, menstrual health, gender, social norms, empowerment

## Abstract

Despite its cultural and biological importance, limited knowledge about menstruation and cultural taboos in many contexts mean that menarche often brings fear and stigma. In Ethiopia, the context of this paper, lack of knowledge and the stigma around menstruation create challenges for adolescent girls related to menstrual hygiene management and their reproductive health more broadly. This paper uses a cluster-randomized controlled trial (cRCT), with 97 communities (*kebeles*) randomly assigned to treatment or control, to assess the impact of a gender-transformative life-skills intervention [Act With Her-Ethiopia (AWH-E)] on the menstrual health literacy of very young adolescent girls and boys (10–14) in two diverse regions of Ethiopia (South Gondar, Amhara and East Hararghe, Oromia). The evaluation employs a longitudinal mixed-methods design, with baseline data collected in late 2017/early 2018 and follow-up data collected in late 2019/early 2020. Quantitative surveys were undertaken with 2,492 very young adolescents and their primary caregivers, and complemented by qualitative interviews with 291 adolescents and their caregivers, as well as 96 key informants at community and district levels. Our quantitative findings highlight large and statistically significant improvements on norms around menstruation, knowledge about menstruation and biological function, and knowledge and behavior related to menstrual hygiene management, but with important differences by location and gender. Qualitative findings further unpack these gender and regional differences, highlighting the importance of adapting programming to the local context and, where possible, connecting to other health and gender initiatives. This analysis helps fill the evidence gap on “what works” to improve menstrual health literacy in rural low- and middle-income contexts.

## Introduction

Adolescence is increasingly recognized as a ‘critical period' for development (1) and is a time of rapid physical transformation. For girls, menarche is a milestone that can have far-reaching impacts on their daily lives. In low- and middle-income countries (LMICs) in particular, menarche often symbolizes the transition to adulthood, and indicates readiness for marriage and childbearing (2). Despite its cultural and biological importance, limited knowledge about menstruation and cultural taboos mean that menarche often brings fear and stigma (3). This stigma connects to broader restrictive gender norms that often keep girls home from school, limit their mobility, and set expectations around appropriate behavior (4). By stifling menstrual health literacy, this stigma not only leads to health risks from unhygienic menstrual hygiene management (MHM) practices, but also decreases access to education and limits fertility awareness and understanding of reproductive health (3).

In Ethiopia, the location of this study, girls often have no knowledge of menstruation prior to reaching menarche (5). This lack of knowledge and the stigma around menstruation mean that adolescent girls face numerous challenges related to their reproductive health (6). In rural and conservative areas in particular, girls are at greater risk of child marriage and sexual violence when they reach menarche, as menstruation is seen as an indicator of sexual maturity and readiness to marry (7). Lack of access to water and inadequate sanitation result in additional challenges for girls' MHM and, along with being teased by peers, can mean that girls miss school or drop out altogether (6).

One cost-effective intervention with the potential for improving menstrual health literacy for girls and boys in LMICs is life-skills programming. The life-skills club format provides structured and safe educational spaces grounded in a defined curriculum guided by a near-peer mentor with whom adolescents can build a trusted relationship (8, 9). Life-skills programmes generally seek to build skills and behaviors that facilitate communication, decision-making, emotion management, relationship development, and overall success in life (10). More recently, programmes have increasingly aspired to be “gender transformative”– that is, to shift gender roles to be more equitable by challenging norms, attitudes and gendered systems, and by including men and boys (11–13).

One intervention that aims to improve menstrual health literacy is Act With Her-Ethiopia (AWH-E)–a gender-transformative life-skills intervention that targets very young adolescents in two diverse regions (Amhara and Oromia). It involves a 10-month safe spaces curriculum-based group programme for girls aged 11–13, with additional programming for boys and others who influence adolescents' lives (including parents, schools, community leaders and other community members), as well as system-strengthening initiatives. The programme aims to help girls and boys build skills and a supportive network to help them navigate the transition from childhood to adulthood, and to support enabling environments for gender norm transformation. One of the programme's focus areas is to tackle gender norms around menstruation and improve menstrual health literacy.

This paper uses a cluster-randomized controlled trial (cRCT) to assess the impact of the AWH-E initiative on MHM and menstrual health literacy among very young adolescents in Ethiopia. Communities (*kebeles*) were randomly assigned to receive the intervention: 58 *kebeles* assigned to treatment and 39 assigned to control. The evaluation employs a longitudinal mixed-methods design, with baseline data collected in late 2017/early 2018 and midline data collected in late 2019/early 2020. Quantitative surveys were undertaken with 2,492 very young adolescents (10–14 years) and their primary female caregivers (or male caregiver if no female was available for the survey), and complemented by qualitative interviews with 291 adolescents and their caregivers, as well as 96 key informants at community and district levels (including programme mentors, health extension workers, community leaders and public health officials). This analysis will help fill the evidence gap on ‘what works' to improve menstrual health literacy in rural LMIC contexts, and in particular for the very young adolescent cohort, where girls are approaching the onset of puberty (rather than already past it). Data on menstruation experiences among girls of this age group is especially limited, given that demographic and health surveys–where they include a menstruation module–are administered to girls and women aged 15–49 years only (14).

“Health literacy” refers to people's ability to understand, obtain and process health information and services (15). Eschler et al. [(16), p. 1] take this as their starting point and define menstrual literacy as “a baseline of knowledge and skills for understanding anatomical and biological facts of menstruation, caring for the menstruating body, and completing menstrual care tasks.” In this paper, we use this definition, and draw on the Gender and Adolescence: Global Evidence (GAGE) conceptual framework to guide our analysis. Informed by the work of Sen (17, 18) and Nussbaum (19), this framework adopts a capabilities approach to emphasize that investments in adolescents must support their development in multidimensional and interconnected physical, psycho-emotional, cognitive and social domains, across the second decade of life and beyond. In the case of menstrual health and wellbeing, it calls for a focus firstly on the individual-level information, support and supplies that adolescent girls need to manage menstruation. The framework also underscores the importance of investments to shift social norms within the family, community and schools to tackle discrimination and stigma and to ensure adequate support for girls so that they can develop their full human capabilities (20).

The remainder of the paper is structured as follows. Section Contextualizing menstrual health literacy and the effects of related interventions further contextualizes menstrual health literacy and the effects of related interventions in LMICs more broadly, and in Ethiopia specifically, summarizing the evidence base on interventions to improve menstrual health literacy. Section Materials and methods details the materials and methods used by the study, while section Results summarizes the mixed-methods findings by region. Section Discussion provides our concluding discussion.

## Contextualizing Menstrual Health Literacy And The Effects Of Related Interventions

### Menstrual Health Literacy in LMICs and in Ethiopia

Many girls in LMICs start menstruation uninformed and unprepared (5). A number of studies find that its physiological basis is not well-understood by girls, and where they do possess knowledge, it is not factual, with many girls still believing menstruation is a curse (21–23). Gultie et al. (24) find that two-thirds of adolescent girls interviewed in Ethiopia's Amhara region believe that menstruation is unnatural; Belayneh and Mekuriaw (25) find that 68% of the adolescent girls in their research in southern Ethiopia have poor knowledge about menstruation, with over half believing that disease is a cause of periods. Knowledge gaps and misconceptions such as these leave girls ill-prepared for when menstruation commences, and exacerbate their fears and anxieties [(5), p. 628].

Menstruation is often stigmatized and perceived as polluting, and this perception shapes girls' knowledge about and experiences of menstruation (3). Drawing on work in Tigray, in Ethiopia, Wall et al. (26) suggest that such perceptions are reinforced by a commonly held belief that being out in direct sunlight can lead girls and women to become unwell–keeping ‘the subject of menstruation “in the shadows,” both figuratively and literally'. Also in Ethiopia, Abita et al. (27) find that even girls who were prepared for menarche were very distressed when it happened, with one describing it as being akin to having a disease.

The stigma of menstruation also has consequences for girls' educational outcomes. Studies in Ethiopia have found that around half of girls report regularly missing days of school when menstruating due to factors such as shame and fear of teasing, as well as discomfort (26, 28–30). In north-west Ethiopia, Tegegne and Sisay (31) find that anxiety about blood leaking onto clothing also makes girls miss school or feel anxious about being in class, which affects their performance. Two-thirds of women and girls interviewed by Wall et al. (32) in Tigray region stated that there are various social, religious and practical expectations and restrictions girls have to observe when menstruating, with 22% of women and 11% of men believing that girls should not attend school when menstruating.

Talking about menstruation is also seen as socially unacceptable in many contexts, making it difficult for girls to access information (33–37). Several studies find that this perception of menstrual topics as not appropriate for discussion makes it difficult for girls to either access accurate information or seek support [(3), p. 17]. For many Ethiopian girls, their mother is their main source of information on menstruation: Abita et al. (27) in north-west Ethiopia finds that 63% of girls identify their mothers as their first source of knowledge about menstruation, while an earlier study in the same region (28) also found that mothers were the leading source of information for girls, followed by peers. However, it also appears that mothers themselves often lack accurate knowledge about menstrual health (5). Nevertheless, Shibeshi et al. (38) find that receiving knowledge about menstruation from one's parents prior to menarche is positively associated with good MHM practices, suggesting that Ethiopian girls who are informed about menstruation are more confident in seeking support, as well as obtaining sanitary pads and other items.

Several studies find that most girls do not learn about menstruation until they begin menarche^1^. In some contexts, this is because it is perceived that they will not understand it until they experience it (3). However, puberty and the physiological changes it brings is also often linked with sexual development and behavior, with menarche often signifying that girls are now sexually available (35, 40–44). In research by Wall et al. (32) in Tigray, half of women and girls interviewed believed that menarche was the marker of sexual maturity or fertility. Sommer et al. (45) find that girls in Ethiopia were fearful of menarche because their parents had instilled in them its connection with sexuality, or even that being sexually active can bring on menarche. The association between reaching sexual maturity and menarche also means that informing younger adolescents about it may be perceived as inappropriate. Yet Coast et al. (46) find that younger adolescents (aged 10–14) in LMICs are inadequately prepared for menstruation. Holmes et al. (15) suggest there is a need for school-based education programmes to address knowledge gaps.

### Gender-Transformative Approaches and Interventions to Improve Menstrual Health Literacy

Holmes et al. (15) observe that few interventions address menstrual health knowledge, with work on menstrual health in LMICs largely focusing on menstrual hygiene instead. They differentiate between “software” (educational) and “hardware” (material) interventions to support hygiene practices (47). However, because of the connection that is generally perceived between menarche and the transition to adulthood, Geertz et al. (48) emphasize that girls' experiences with menstruation are inextricably linked to the gender norms that increasingly restrict their lives as they reach puberty, while new gender inequities can arise as a result of social restrictions imposed on girls during menstruation (49). Gender has thus been increasingly recognized as an important social determinant of health, prompting a shift within health interventions to approaches that seek to address harmful gender norms (50). Gender-transformative approaches that engage with these norms thus hold promise for addressing menstrual health literacy gaps, and are seen as key to achieving the Sustainable Development Goals (SDGs) (51).

Implementing gender-transformative approaches to menstrual health is complex because of the multiplicity of factors involved. For example, Hennegan et al. (3) suggest that because menstrual experiences are shaped by sociocultural context as well as resource constraints, interventions need to address girls' social support, the material environment and wider restrictive norms and beliefs, as well as improving girls' menstrual health knowledge. Levy et al. (12) note the need to also consider intersectionality in the design of interventions because race, religion, geographic location and disability will impact girls' experiences of gender norms and inequalities. This reflects Heise et al. (51) suggestion that “clustered deprivations” have consequences for health, and thus there is a need to go beyond simple binaries in understanding barriers to better outcomes. For example, research in Ethiopia by Shibeshi et al. (38) finds disparities between rural and urban girls with regards to MHM practices, with girls in rural areas less likely to have good practices. This disparity is driven both by the reluctance of parents in rural areas to discuss reproductive health issues with their daughters, and the availability of information in urban areas (through the media) enabling even those whose parents will not discuss the topic to access information and improve their menstrual health literacy [(38), p. 10].

Embedding a gender-transformative approach within life-skills education offers one avenue for interventions. Comprehensive sexuality education (CSE) in schools, starting before adolescent girls reach puberty, is frequently advocated as key to improving menstrual health literacy in LMICs (22, 37, 52, 53). However, there remain barriers to the implementation of gender-transformative CSE interventions. Le Mat et al. (54), for example, show that in the Ethiopian context, CSE lacks engagement with issues of gender inequality such as gender-based violence. This is due to political dynamics around the adoption and reformulation of donor-driven CSE, which have restricted curricula to promoting development-related health outcomes rather than addressing gender norms. Levy et al. (12) also find that many programme activities with so-called gender-transformative intent that aim to improve health-related outcomes do so by primarily shifting individual and interpersonal indicators related to agency, and attitudes and behaviors on gender norms. However, because gender norms and inequality are structural challenges, this approach does not necessarily lead to systemic change (55).

Indeed, previous interventions demonstrate an emphasis on improving knowledge, but have often been unable to address the structural norms that make menstruation such a challenging experience for girls, even when they do have better menstrual health literacy. For example, improved knowledge, beliefs and hygiene practices resulted from education interventions in Uganda (56), Bangladesh (57) and Nepal (58). Although RCTs are less common, a cRCT in Kenya explored the impact on knowledge and attitudes of participating in reproductive health education classes over 25 sessions, delivered by trained facilitators during girls-only health clubs. The authors find that girls who participated felt more positively about menstruation, knew more about sexual and reproductive health, had more equitable gender norms, and were more self-confident by the end of the classes than those who did not Austrian et al. (59). However, the intervention did not engage with the wider community to address drivers of poor knowledge or discriminatory gender norms.

Interventions in Ethiopia have also tended to focus on knowledge about menstruation, sometimes aiming to improve other outcomes. Blake et al. (60) analyze an RCT piloting books on puberty and menstruation for girls in Oromia region, and also find a significant improvement in knowledge and attitudes around menstrual health. However, the authors also emphasize that investments in creating a more gender-equitable physical and social environment would augment the effects of this individual-level intervention (ibid.). This echoes the review of menstrual health literacy in LMICs by Holmes et al. (15), who find that there is an absence of coordinated cross-sectoral effort by stakeholders to address both the factors shaping menstrual health literacy as well as literacy itself.

## Materials and Methods

### Setting

**Figure 1:**
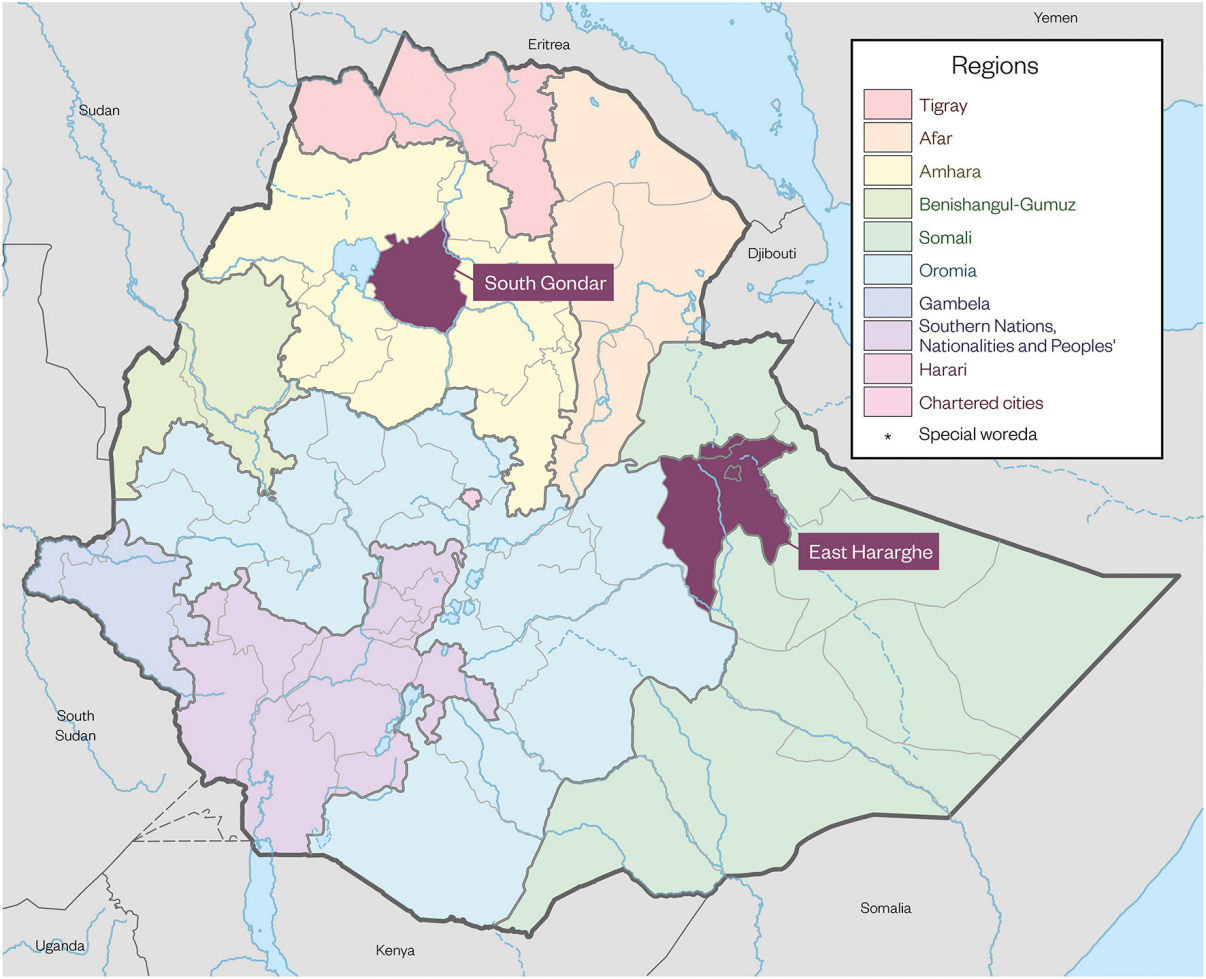
Map of research sites.

The setting for this study is two densely populated zones in Ethiopia–East Hararghe in Oromia region and South Gondar in Amhara region (see Figure 1).

East Hararghe zone residents are predominantly ethnic Oromos and the majority of the population are Muslims. East Hararghe is partly highland and partly semi-lowland, and favorable for food and cash crop production and animal husbandry. Most of the *woredas* (districts) in the zone are known for the production of cash crops such as khat and groundnuts, which are mainly exported to Somalia, Somaliland, Djibouti and the Middle East. Due to recurrent drought, land fragmentation, and large average household size, many people are poor and dependent on food assistance for most of the year. Due to entrenched gender and social norms, child marriage and female genital mutilation/cutting (FGM/C) are still prevalent in the area, affecting the health and education of many girls.

South Gondar is home to the Amhara people, most of whom follow the Ethiopian Orthodox Church. Most of the districts in this zone are highland, with crop production for food consumption the main livelihood source of most rural households. Due to drought and land shortages, many Amhara adolescents migrate to urban centers or to cash crop-producing areas in search of temporary employment. Social problems such as alcoholism, gang violence, gender-based violence and child marriage are major challenges for the communities in this area.

### Sample and Study Design

Communities (*kebeles*) eligible for GAGE research in East Hararghe and South Gondar were chosen on the basis of two key considerations: (1) a review of existing data and evidence on adolescents and gender in Ethiopia, which highlighted the importance of understanding both the economic and social drivers that underpin disadvantage (61–63); and (2) capacity (including operational presence and experience, and an absence of security concerns) on the part of the non-governmental organization (NGO) implementing partners, Pathfinder International (in Amhara) and CARE Ethiopia (in Oromia), which would provide adolescent-focused programming in these zones. From 235 eligible communities, 155 were randomly chosen for participation in the GAGE research. In late 2017, a door-to-door household listing exercise was conducted in the research communities to identify adolescents aged 10–12 living there. Nearly 4,000 adolescents were randomly sampled from these lists for GAGE study recruitment. Around this same time, and prior to the start of baseline data collection (at the individual adolescent and household levels), the 155 *kebeles* were randomized into control and four hypothetical treatment arms (stratifying on *woreda* and marginalization status). Randomization was done by creating a random number in Microsoft Excel and then ordering by *woreda*, marginalization status and then the random number.

The analysis in this paper focuses on adolescents in the subset of communities that were randomly allocated to the control group and two of the treatment arms–those that received the full Act With Her–Ethiopia (AWH-E) programme (described below), and communities that received a slightly simpler version of this programme (focused on adolescents and their families)^2^. The quantitative analysis sample thus includes 2,492 adolescents (1,431 girls and 1,061 boys) aged 10–12 at recruitment in late 2017, living in 97 *kebeles* across East Hararghe and South Gondar zones. Baseline quantitative data was collected for nearly all of these adolescents prior to the launch of programme implementation in early 2018, including a rich set of information on household demographics, wealth and vulnerability, as well as adolescent attitudes and behaviors.

In early 2019, Pathfinder (in South Gondar) and CARE (in East Hararghe) launched AWH-E programming in communities allocated to the appropriate study treatment arms. AWH-E was designed to facilitate adolescent transitions to adulthood via improvements across the six GAGE capability domains (education, bodily integrity, health, psychosocial wellbeing, voice and agency, and economic empowerment). Project activities fell into three main categories: all AWH-E communities received (1) weekly curriculum-based programming with adolescent girls and boys aged 11–13 and six group meetings for their parents over 10 months, and (2) facilitated community conversations on discriminatory gender norms and how to tackle them; a subset of communities also received (3) interventions to strengthen community-level systems, including the construction of MHM facilities in school. The community-level system strengthening work lasted for up to 24 months.

AWH-E developed project-specific curricula for boys and girls, which comprehensively address key topics across the six GAGE domains, including information on menstrual health and puberty. In particular, the girls' curriculum included sessions on puberty, sexual and reproductive health, exercise and nutrition, decision making and negotiation tactics, and financial skills, among many others. Girls of eligible age participated in weekly curriculum-based, near-peer mentor-led group sessions (40 sessions in total). Near-peer male mentor-led groups for boys ran concurrently, meeting approximately twice a month (18 sessions in total). Topics covered in the boys' and girls' groups were temporally aligned, and four sessions brought the boys' and girls' groups together. The AWH-E consortium also developed a parents' guide, including six sessions to orient parents (or any primary caregiver of the adolescents involved) to the topics covered in the adolescent curricula and to help them create a supportive environment for their adolescent. The community-level systems-strengthening activities focused on: (1) establishing locally led mechanisms for discussing social norms in ways that initiate shifts over time (including past the project timeline); and (2) enhancing local capacity for social accountability. One proposed focus of community-level systems (and services) strengthening was enhanced MHM. (For more detail on the interventions, see: www.pathfinder.org/projects/act-with-her).

A follow-up quantitative survey was conducted as part of the GAGE research in late 2019 and early 2020 in the treatment and control communities when the adolescents were aged 12–15. This follow-up was conducted approximately 10 months after the launch of programming, shortly after the adolescent and parent group meetings had been completed. The survey collected information on adolescent outcomes across a range of topics, including questions on knowledge related to menstruation (e.g., knowledge of menstruation frequency; knowledge that menstruation means girls can get pregnant, etc.) as well as on MHM. More than 88% of adolescents (*n* = 2,204) were successfully surveyed.

This quantitative data collection was complemented by in-depth qualitative research (*n* = 291) with adolescents (in the control and treatment arms) and other key stakeholders (such as parents, mentors and government officials) in order to better understand some of the emerging patterns and mixed pictures painted by the survey findings.

The RCT design and data collection tools were approved by the relevant local and international ethics bodies: the George Washington University Committee on Human Research's Institutional Review Board (071721); the Overseas Development Institute Research Ethics Committee (02438); the Ethiopian Development Research Institute (EDRI/DP/00689/10); the Addis Ababa University College of Health Sciences Institutional Review Board (113/17/Ext); and the Amhara and Oromia regional health bureau ethics committees. Consent (written or verbal) was obtained from caregivers and married adolescents, and written or verbal assent was obtained for all unmarried adolescents under 18 years. There was a robust protocol for referral to services, tailored to the realities of adolescents in the diverse study sites.

### Quantitative Measures

The quantitative analysis in this paper focuses on a series of menstrual health, hygiene and management measures that were collected as part of the adolescent follow-up survey (available at https://www.gage.odi.org/publication/ethiopia-round-2-survey-2019-2020/). The survey uses validated measures as well as measures designed by the broader GAGE consortium of subfield experts. Surveys were translated into regional-specific languages and then back-translated for confirmation of original question integrity.

For girls and boys alike, we explore a set of questions on knowledge about menstruation and pregnancy that were covered in the AWH-E curriculum. These measures include: (i) an indicator for knowing that girls usually reach puberty before boys; (ii) an indicator for knowing that menstruation typically occurs approximately once per month; and (iii) an indicator for knowing that girls who have reached menarche can become pregnant. For girls only, we also measure (iv) an indicator for having ever talked about menstruation with their mother or female guardian.

Among girls who had reached menarche by the time of the follow-up survey (only 13% of the girls in our sample), we additionally focus on a set of measures of menstrual hygiene practices as follows: (i) a binary self-report of whether normal (daily) activities are affected during menstruation (such as social, religious or employment activities); (ii) a binary self-report of whether improved MHM methods are used (such as sanitary pads or other modern sanitary products); and (iii) a binary self-report of whether appropriate disposal of MHM products is practiced at home (such as latrine disposal or burning). Given the small percentage of girls in our sample who had reached menarche, the findings in this domain should be interpreted as suggestive.

### Data Analysis

The quantitative analysis utilizes multivariate regressions to assess intention to treat programme impacts across the set of outcomes described above related to menstrual health literacy and MHM 10 months after the start of the programme. The sample includes all adolescents who were randomly sampled for recruitment to the GAGE study in the control and two treatment (AWH-E, and AWH-E minus community systems-strengthening) sites, and were surveyed in the follow-up data collection^3^. Specifically, we estimate the following reduced form linear regression model:


(1) $$\begin{array}{r} y_{ic}=\alpha_{1}+\beta_{1}T_{c}+{X^{\prime }}_{ic}\beta_{2}+\varepsilon_{ic} \end{array}$$

where *y*_*ic*_ is the outcome of interest for adolescent i in community c, and *T*_*c*_ is a binary indicator for baseline residence in an *AWH-E* community as defined above. *X*_*ic*_ includes measures used in the treatment randomization, as well as a rich set of baseline controls. The former includes strata indicators (where strata combine *woreda* and community marginalization status), adolescent age (in years), and an indicator for whether there were multiple eligible adolescents in the household at the baseline data collection. The rich baseline controls include household size, an indicator for literacy of the household head, an indicator for female headed households, a household asset index, an indicator for households that have ever received Productive Safety Net Programme (PSNP) benefits, and follow-up survey month indicators^4^. These controls were pre-specified at the time of trial registration (64), and are included either because they were part of the randomization process or because they are strongly predictive of the outcomes of interest, thus improving the precision of the impact estimates (65). All regressions are weighted to maintain initial population proportions, and standard errors are clustered by community (*kebele*). It is worth noting that the literature suggests that this linear model (used for ease of interpretation) performs as well as others (66, 67).

The regression analysis is complemented by qualitative interviews that were transcribed, translated and coded thematically in MAXQDA, a qualitative data analysis software package. The codebook and analysis were informed by the GAGE conceptual framework (see Introduction), and paid particular attention to changes brought about by programming in: (1) beliefs and norms related to menstruation; (2) knowledge about menstruation facts; (3) knowledge about MHM; and (4) the extent to which this knowledge could be applied locally given infrastructural, socioeconomic and cultural factors. The analysis focused on changes among adolescent girls and boys, their primary caregivers, programme mentors and community service providers.

## Results

### Baseline Balance and Attrition

Before proceeding with our findings on the impact of AWH-E programming, we first assess baseline balance between the treatment and control communities (Tables 1A,B) and attrition (Table 2). The sample is well balanced at baseline on a set of demographic characteristics, with the only exception being whether the household head is female for the male sub-sample in both regions. Across a total of 54 comparisons, 3 are statistically significant at 95% confidence, which is what one would expect by ‘statistical chance'. This finding suggests that the randomization was conducted successfully.

**Table 1A:** Baseline descriptive statistics for full sample.

	**Overall**	**Females**			**Males**		
		**Control**	**Treatment**	**SigDiff**	**Control**	**Treatment**	**SigDiff**
=1 recruited in East Hararghe	0.529	0.520	0.511		0.563	0.526	
=1 recruited in South Gondar	0.471	0.480	0.489		0.437	0.474	
Age at recruitment	10.9	10.9	10.9		11.0	10.9	
	(0.8)	(0.8)	(0.8)		(0.8)	(0.8)	
=1 if multiple eligible adolescents in household	0.053	0.063	0.066		0.055	0.032	
Household size	6.4	6.3	6.4		6.4	6.4	
	(1.8)	(1.8)	(1.9)		(1.9)	(1.7)	
=1 if household female headed	0.131	0.127	0.133		0.177	0.099	***
=1 if household head literate	0.326	0.311	0.322		0.309	0.351	
Household asset index	3.69	3.81	3.70		3.58	3.69	
	(2.08)	(2.10)	(2.13)		(2.04)	(2.06)	
=1 household is a PSNP recipient	0.305	0.360	0.294		0.307	0.276	
Number of observations	2,492	584	846		427	635	

This table summarizes key baseline characteristics of the full sample. Each cell contains a sample mean (weighted to maintain initial population proportions), and the standard deviation is provided in parentheses for continuous measures. The “SigDiff” column notes a statistically significant difference at p < 0.1 (*), p < 0.05 (**), and p < 0.01 (***). The asset index takes a value between 1 and 9 indicating the decile of household assets in relation to the rest of the GAGE sample in Ethiopia (based on a series of indicators for 16 different measures of household durables).

**Table 1B:** Baseline descriptive statistics for each region.

	**Overall**	**Females**			**Males**		
		**Control**	**Treatment**	**SigDiff**	**Control**	**Treatment**	**SigDiff**
**Panel A: East hararghe sample**							
Age at recruitment	10.9	10.9	10.9		11.0	10.9	
	(0.8)	(0.9)	(0.8)		(0.8)	(0.8)	
=1 if multiple eligible adolescents in household	0.079	0.100	0.102		0.082	0.044	*
Household size	7.0	6.9	7.1		7.0	7.0	
	(1.8)	(1.8)	(1.8)		(1.8)	(1.7)	
=1 if household female headed	0.111	0.115	0.105		0.162	0.075	***
=1 if household head literate	0.283	0.249	0.316		0.253	0.300	
Household asset index	3.41	3.36	3.42		3.29	3.52	
	(1.92)	(1.86)	(1.99)		(1.82)	(1.98)	
=1 household is a PSNP recipient	0.305	0.383	0.294		0.300	0.264	
Number of observations	1,282	300	434		220	328	
**Panel B: South Gondar sample**							
Age at recruitment	10.9	11.0	10.9		11.0	10.9	
	(0.8)	(0.8)	(0.8)		(0.8)	(0.8)	
=1 if multiple eligible adolescents in household	0.023	0.023	0.028		0.020	0.020	
Household size	5.7	5.7	5.7		5.6	5.8	
	(1.6)	(1.6)	(1.6)		(1.6)	(1.5)	
=1 if household female headed	0.153	0.141	0.162		0.197	0.126	***
=1 if household head literate	0.374	0.379	0.328		0.381	0.409	
Household asset index	4.01	4.29	3.99		3.95	3.88	
	(2.21)	(2.24)	(2.23)		(2.25)	(2.13)	
=1 household is a PSNP recipient	0.305	0.336	0.294		0.315	0.289	
Number of observations	1,210	284	412		207	307	

This table summarizes key baseline characteristics of the sample. Panel A includes those recruited in East Hararghe zone, and Panel B includes those recruited in South Gondar zone. Each cell contains a sample mean (weighted to maintain initial population proportions), and the standard deviation is provided in parentheses for continuous measures. The ‘SigDiff' column notes a statistically significant difference at p < 0.1 (*), p < 0.05 (**), and p < 0.01 (***). The asset index takes a value between 1 and 9 indicating the decile of household assets in relation to the rest of the GAGE sample in Ethiopia (based on a series of indicators for 16 different measures of household durables).

**Table 2:** Attrition analysis (Outcome =1 if interviewed in follow-up survey).

	**Full sample**				**East Hararghe sample**				**South Gondar sample**			
	**Female**		**Male**		**Female**		**Male**		**Female**		**Male**	
	**(1)**	**(2)**	**(3)**	**(4)**	**(5)**	**(6)**	**(7)**	**(8)**	**(9)**	**(10)**	**(11)**	**(12)**
=1 if in AWH-E community	0.007	−0.002	−0.022	0.081	−0.019	−0.086	−0.031	0.016	0.035*	0.057	−0.011	0.146
	(0.028)	(0.103)	(0.028)	(0.085)	(0.051)	(0.176)	(0.049)	(0.151)	(0.020)	(0.114)	(0.019)	(0.115)
=1 if baseline location in East	−0.006	−0.020	−0.007	−0.029								
Hararghe	(0.047)	(0.042)	(0.054)	(0.060)								
Age at the time of GAGE listing	−0.017	−0.015	−0.022*	−0.023*	−0.030	−0.029	−0.030	−0.031	0.000	−0.001	−0.013	−0.014
	(0.013)	(0.012)	(0.012)	(0.012)	(0.020)	(0.020)	(0.019)	(0.020)	(0.013)	(0.013)	(0.014)	(0.013)
Baseline household size		0.023*		0.018**		0.013		0.009		0.031**		0.029**
		(0.012)		(0.009)		(0.018)		(0.015)		(0.013)		(0.011)
=1 if baseline household head is		0.061**		0.058*		0.027		0.053		0.106***		0.065
female		(0.027)		(0.030)		(0.041)		(0.044)		(0.036)		(0.046)
=1 if baseline household head		0.044		0.063**		0.015		0.074*		0.083**		0.053
literate		(0.032)		(0.028)		(0.057)		(0.042)		(0.038)		(0.032)
Baseline decile of household		−0.005		0.001		0.005		0.011		−0.013*		−0.006
assets (standardized)		(0.008)		(0.010)		(0.014)		(0.019)		(0.008)		(0.008)
=1 if household had received		0.019		−0.024		0.029		−0.048		−0.006		−0.002
PSNP benefits		(0.032)		(0.034)		(0.050)		(0.053)		(0.037)		(0.034)
F-test *p*-value		0.553		0.386		0.821		0.703		0.050		0.318
Control mean (outcome)	0.861	0.861	0.895	0.895	0.821	0.821	0.855	0.855	0.904	0.904	0.947	0.947
Observations	1,430	1,430	1,062	1,062	734	734	548	548	696	696	514	514

This table presents results from regressions for an indicator for the CR being surveyed in the GAGE round 2 survey, including coefficient estimates and standard errors (in parentheses). All regressions include as covariates an indicator for households that had multiple adolescents eligible for the study and sampling block fixed effects. The even columns additionally include a set of interactions for each ‘rich' control and the treatment measure. The F-test tests joint statistical significance across the treatment measure and all of these interactions. Regressions are weighted to maintain initial population proportions, and standard errors are clustered by kebele. ^*^ denotes statistical significance at p < 0.1, ^**^ at p < 0.05, ^***^ at p < 0.01. The asset index takes a value between 1 and 9 indicating the decile of household assets in relation to the rest of the GAGE sample in Ethiopia (based on a series of indicators for 16 different measures of household durables).

Our sample was approximately evenly split between East Hararghe and South Gondar (Table 1A). At baseline, on average, adolescents were 10.9 years old and came from households with 6.4 members. Just under a third of households (30.3%) received benefits from the PSNP and 13.1% were headed by a female. In 32.6% of households, the household head was literate. There were some important differences across the two locations (Table 1B), with literacy rates higher in South Gondar (37.4 vs. 28.3%) and poverty rates lower (asset index of 4.01 vs. 3.41).

In terms of attrition (Table 2), we successfully surveyed 86.1% of girls and 89.5% of boys at round 2, thus attrition rates are <15% for both groups. We see no overall differential attrition between AWH-E and control communities for either gender, and see only a small and marginally significant increase in the likelihood of surveying girls in the treatment as compared to the control groups in South Gondar (3.5 percentage points, *p* < 0.1). While we are more likely to survey girls and boys from larger households, and girls in households where the household head is literate, there is no differential likelihood of surveying treatment and control girls or boys based on baseline characteristics across most samples. There is some indication of differential attrition by baseline characteristics for girls in South Gondar (*p* = 0.05), and results should be interpreted with this in mind. The limited evidence of differential attrition reinforces the causal interpretation for our quantitative estimates.

### Regional Impact of AWH-E Programming

Given the vast differences in South Gondar and East Hararghe discussed above, we structure our mixed-methods findings by region. We use the definition of menstrual literacy to organize our findings across the following domains: (1) changes in norms around menstruation (mixed methods); (2) changes in knowledge about menstruation and biological function (mixed methods); (3) changes in knowledge and behavior related to MHM (mixed methods); and (4) ability to apply this knowledge to local contexts (qualitative).

#### South Gondar, Amhara

##### Changes in Norms Around Menstruation

Quantitative findings show that adolescent girls in AWH-E communities were significantly more likely to report talking to their mothers about menstruation (22 percentage points over a base of 14.5%, *p* < 0.001, Table 3, Panel B). This impact is large–corresponding to a 151% increase. Note, however, that this still means that only a little over one in three adolescent girls in treatment communities talk to their mother about menstruation. Our qualitative data provides evidence that this increase in communication builds from a widespread shift in norms due to AWH-E programming, norms that historically stigmatized menstruation and associated it with a curse. As a male mentor from Community A noted, the programme curriculum has placed a strong emphasis on dispelling these stigmatizing norms and reaching girls prior to the onset of menarche:

*Previously, menstruation was considered as a curse in our locality. Girls would never even prepare coffee for their parents if they were menstruating. Even more, if a school-going girl is menstruating, no one goes to school with her, others will isolate her, and she will be dissociated from her friends, and may become absent from school. However, there is a topic [in the AWH-E curriculum] about menstruation, and children got some lessons on it. Most of them are still too young … only three or four girls may have reached menarche. Therefore, the training makes them aware and prepares them for when they reach it. The content also helped them consider menstruation as natural for all women*.

(Male mentor, Community A, South Gondar).

**Table 3:** AWH-E Impacts on female menstrual health practices and knowledge.

	**(1)**	**(2)**	**(3)**	**(4)**	**(5)**	**(6)**	**(7)**
	**Indic talks to mother about menstruation**	**Indic activ not affected by menst**	**Indic impr menst mgmt**	**Indic approp disposal**	**Knowledge - indic girls reach puberty first**	**Knowledge - indic menstruation frequency**	**Knowledge - indic menarche allows pregnancy**
**Panel A: East Hararghe sample**							
=1 if in AWH-E community	0.046*	0.102	0.262**	0.042	0.002	0.102**	0.090**
	(0.023)	(0.075)	(0.100)	(0.086)	(0.040)	(0.044)	(0.037)
Control mean	0.079	0.785	0.195	0.361	0.725	0.351	0.586
Observations	493	112	112	92	607	605	607
**Panel B: South Gondar sample**							
=1 if in AWH-E community	0.222***	−0.060	0.180	−0.104	0.016	0.158***	−0.052*
	(0.042)	(0.154)	(0.166)	(0.184)	(0.035)	(0.038)	(0.027)
Control mean	0.145	0.952	0.466	0.463	0.287	0.584	0.810
Observations	602	50	49	47	645	644	645

This table presents results from regressions for adolescent female outcomes, including coefficient estimates and standard errors (in parentheses). All regressions include as covariates adolescent age at the time of recruitment, an indicator for households that had multiple adolescents eligible for the study, sampling block and survey month fixed effects. In additional, all regressions control for household size, a household asset index, an indicator for the household head being literate, an indicator for the household head being female, and an indicator for the household having ever received PSNP benefits. Regressions are weighted to maintain initial population proportions, and standard errors are clustered by kebele. ^*^ denotes statistical significance at p < 0.1, ^**^ at p < 0.05, ^***^ at p < 0.01.

Echoing these comments, adolescent girls who participated in the programme commonly noted that the curriculum had helped them become aware of menstruation as a natural biological phenomenon, or even a blessing, about which they should not feel ashamed. For example, an 11-year-old girl from Community A noted: ‘*I have now better awareness about menstruation and understand that it is a normal phenomenon in which girls shouldn't feel humiliated.'*

The qualitative data also indicates that this shift in norms is being shared more widely among the community. Some mentors noted that girls participating in their sessions had passed on learning about menstruation to their mothers:

*The girls are helping the mothers to change their views*. *Mothers say to me we are happy that you have helped in preparing our daughters by teaching them precautionary measures before they experience their period, and that their daughters persuaded them that this will help them to pursue their education*.

(Female mentor, Community C, South Gondar).

Another female mentor, from Community A, similarly explained that engaging with mothers about issues related to the changes adolescents undergo during puberty (including menstruation) was encouraging norm change among caregivers too. While not all mothers were immediately convinced, within the duration of the programme (10 months), a tipping point was reached within the group whereby participants who were initially resistant had shifted their beliefs once they saw that the majority were doing so:

*In one of the training sessions, there was a debate on the issue of supporting girls to prepare coffee when they are menstruating … During this debate only one woman opposed girls being able to prepare coffee during their menstruation as it is a curse … She remained rigid [in holding that view] for some two or three months, and then after all the other mothers talked to her, she accepted and she changed her idea of considering menstruation as a curse. She told us that she accepted what the group said and she never wanted to be isolated from other mothers in the session, because all other mothers in the session had changed their perception, and that helped convince her*.

(Female mentor, Community A, South Gondar).

These norm changes were also reflected among boys and men, who explained that the programme has helped boys to reshape their perceptions about menstruation. Boys reported improved attitudes and behaviors, especially that they now refrained from teasing girls when they have their period at school. A 12-year-old boy from Community B explained: ‘*We used to perceive menstruation as something wrong and we used to laugh at girls when they had their period. But I am no longer laughing at girls as I understood it is a normal process.'*

In the same vein, some fathers who were involved in community discussions organized as part of the programme also noted that these had led to important shifts in their views about menstruation:

*Thanks to the programme we are shifting our ideas. There is a norm that forbids a man to eat food at home when he observes menstrual blood on a woman's clothes…he considers it disgusting. At first fathers did not accept this teaching but slowly we are changing*.

(Father of participant in AWH-E, Community A, South Gondar).

##### Changes in Knowledge About Menstruation and Biological Function

We find no significant quantitative impact of AWH-E on knowledge for boys in South Gondar across the three measures (Table 4, Panel B), with overall knowledge ranging from 81% answering correctly that menarche means a girl can now get pregnant, 40.7% knowing the frequency of menstruation, and 65.5% knowing that girls reach puberty earlier than boys (on average). We do, however, see large improvements for girls on an indicator for knowledge of menstruation frequency. Adolescent girls in AWH-E communities are 15.8 percentage points (or 27%, the mean in the control group is 58.4%) more likely to answer this question correctly compared to those in control communities (Table 3, Panel B). We see no significant impact on knowledge that girls reach puberty earlier than boys, and knowledge in this domain is generally low, with only 28.7% in the control group answering this correctly. We also see no significant impact on knowledge that menarche means a girl can get pregnant, but here, knowledge levels are much higher, with 81% of girls answering this correctly.

**Table 4:** AWH-E impacts on male menstrual health knowledge.

	**(1)**	**(2)**	**(3)**
	**Knowledge-indicator girls reach puberty first**	**Knowledge-indicator menstruation frequency**	**Knowledge-indicator menarche allows pregnancy**
**Panel A: East Hararghe sample**			
=1 if in AWH-E	0.029	0.055	0.116***
community	(0.041)	(0.053)	(0.036)
Control mean	0.740	0.231	0.563
Observations	467	466	467
**Panel B: South Gondar sample**			
=1 if in AWH-E	−0.014	0.070	0.012
community	(0.039)	(0.061)	(0.033)
Control mean	0.655	0.407	0.801
Observations	483	481	483

This table presents results from regressions for adolescent male outcomes, including coefficient estimates and standard errors (in parentheses). All regressions include as covariates adolescent age at the time of recruitment, an indicator for households that had multiple adolescents eligible for the study, sampling block and survey month fixed effects. In additional, all regressions control for household size, a household asset index, an indicator for the household head being literate, an indicator for the household head being female, and an indicator for the household having ever received PSNP benefits. Regressions are weighted to maintain initial population proportions, and standard errors are clustered by kebele. * denotes statistical significance at p < 0.1, ** at p < 0.05, *** at p < 0.01.

The qualitative findings further unpack these quantitative impacts for girls, highlighting important shifts in knowledge about biological facts related to menstruation among adolescents who participated in the programme, almost all of whom in the qualitative sample had not yet reached menarche. A 12-year-old girl from Community A explained: ‘*It is a symptom of maturity in which a girl could get pregnant if she has sexual intercourse. A girl could get pregnant if she were raped.'* Some noted that they had already been exposed to information about menstruation at school, in biology class, but that the AWH-E curriculum reinforced and deepened this knowledge, and helped them to internalize messaging about treating it as a natural phenomenon:

*Of course we know about menstruation … but no one in our group has seen it yet … If they are bleeding too much, they will be exposed to anaemia … [The facilitator] told us to go to clinic if the pain is too much or if the flow is heavy. We also shouldn't be ashamed to go to school when seeing menstruation for the first time. Or to teach our sisters if they fear and feel ashamed to go to school because of menstruation … We are taught that menstruation is a natural gift*.

(Girl, aged 11, Community B, South Gondar).

Several mentors noted that non-participants expressed envy toward those who were participating in AWH-E programming because of their access to new information, including on puberty and menstruation; they also noted that non-participants were learning more about the topic through their peers who were participating:

*Trainee girls told me that those non-AWH-E girls feel angry and regret for not getting the chance. However, they also told me that those girls will accept what the trained [participating] girls told them about what they have learnt. These non-AWH-E girls also ask AWH-E girls what they are learning about. Trainee girls told me that they explain they learned about menstruation, HIV and AIDS, and about role model women*.

(Female mentor, Community A, South Gondar).

It is important to note, however, that some adolescent respondents did appear to have some misunderstandings about the menstrual cycle and risk of pregnancy. As a 12-year-old girl from Community B incorrectly noted: ‘*There is a natural method which involves counting the date of the monthly period as well … after her period comes, the next 14 days are safe to have sexual intercourse but after that it's risky.'* Knowledge gains around menstruation were not limited to girls but extended to mothers–regarding both their own menstrual health but also how to support their daughters. A mentor from Community A explained the programme's approach:

*There are girls who are able to prepare pads with used clothes for their mother … The mothers don't mind because we have already discussed with them … We have discussed with mothers by targeting students' mothers. We discuss the menstruation cycle and how it is affecting students' education. We tell them about the prevailing mistaken perception about menstruation that causes girls to drop out of school as they are harassed by others, which in turn forces them to marry early, which can also expose them to fistula. So, parents now have a good understanding about menstruation as well as other harmful practices*.

(Female mentor, Community A, South Gondar).

##### Changes in Knowledge and Behavior Related to Menstrual Hygiene Management

Our quantitative findings in this domain focused on behavior change related to MHM among girls who had reached menarche. This sample size is very small at baseline (50 girls), and results are inconclusive (Table 3, Panel B). Impacts are insignificant across all three measures (indicator for activities not impacted by menstruation, indicator for improved MHM, and indicator for appropriate disposal of sanitary products) and not going in any clear direction. It is worth noting that in the control group, 95.2% of this sample indicated that activities are not impacted by menstruation, with only 46% of girls who had reached menarche in the control group noting either improved MHM or appropriate disposal of products.

While the quantitative survey did not look at knowledge related to MHM, the qualitative findings underscore important changes in knowledge about MHM brought about by AWH-E, emphasizing the fact that this was distinctive from class content offered in school. Indeed, a health extension worker from Community A emphasized that: ‘*I rank managing the menstrual cycle as the most significant change*'. Similarly, a 12-year-old girl from Community B noted that:

*We had some knowledge from school [about menstruation] but nothing about sanitary pads … We are taught to use sanitary pads … or to ask our parents to buy for us sanitary pads when we finish … We know that we have to use it when our period comes and to wash our underwear frequently … Other than that, the teaching is the same*.

Another girl from Community A explained that in her school, teachers had provided more information to older girls once they had experienced their first period, but that it was helpful to have this exposure in very early adolescence so as to prepare younger girls before they reach menarche:

*We were told to use pads or clothes while managing menstrual cycle … It is a spongy material that we saw at school while it was demonstrated by teachers but only to school girls aged 13 or over that experience menstruation … It was given to those girls who belonged to poor households … Pads are not available here, they are bought at Ebenat [district town] … It is during the AWH-E training session that we were given a clear understanding about managing menstruation … It is possible to use strips of cloth or wear shorts … It causes headache as well as discomfort and pain in one's stomach during menstruation …*

(Girl, aged 13, Community A, AWH-E participant).

These qualitative findings provide hope for future impacts on behavior when the sample includes a larger proportion of girls who have reached menarche.

##### Ability to Implement Improved Menstrual Health Management Knowledge in Context

While adolescent girls may get access to information about MHM, their ability to use this knowledge in practice, in resource-constrained rural communities, may be very limited. Our qualitative findings indicate that in South Gondar, female programme participants–both girls and primary female caregivers–gained important practical and actionable knowledge about MHM. A health extension worker from Community C who cooperated with the programme noted as follows:

*After I received the training, I showed mothers how they can easily prepare pads using cloth. These days, girls are [more] free when they experience menstruation, which help them to attend their education without any humiliation. Such behavioral change becomes possible after the intervention of this project. That is why I personally learnt a lot from the project*.

(Health extension worker, Community C).

What was perhaps most innovative about the programme's approach to MHM was the involvement of boys directly in making pads and in the construction of school-based MHM infrastructure (in one community) as an entry point to changing boys' perceptions and behavior toward their female peers around menstruation. The construction enabled girls to exercise hygiene practices if they were menstruating at school, but the change in perceptions allowed them to do so without feeling embarrassed. One boy from Community A explained how he valued this approach to awareness-raising:

*We were trained how to sew and prepare a menstrual pad. In fact, our participation was for a reason: it was not only to offer human resource but also to participate and engage in an attempt to alleviate the problem. It was intended to engage us in the programme so that we can have awareness about the subject*.

(Boy, aged 14, Community A, AWH-E participant).

Another 13-year-old boy from the same community emphasized how he and his programme peers were actively involved in the construction of MHM facilities at his school and the importance of such facilities for female students:

*We actually implemented together with the community. For instance, we have learned about the natural cycle of menstruation. After we learnt about it, we have stopped stigmatising females and started to understand the situation. Therefore, there would be no more school dropout for females due to menstruation. Because everyone understands that it is a natural cycle and [we all] should take part in supporting girls in that situation. To further alleviate the problem, we have prepared the toilet. We built it here in our school compound. Thus, girls can use the toilet to take care of themselves. There is also a sign and a poster, which guide the user on how to use the toilet and keep it clean … The toilet also has a shower facility … There is also a change of clothes for females to change into til their clothes dry*.

(Boy, aged 13, Community A, AWH-E participant).

#### East Hararghe, Oromia

##### Changes in Norms Around Menstruation

Quantitative findings in East Hararghe zone show that girls in AWH-E communities were (marginally) significantly more likely to report talking to their mother about menstruation (4.6 percentage points over a base of 7.9%, *p* < 0.10, see Table 4, Panel A). While the impact is small compared to results in South Gondar, it is from a much lower base, and still corresponds to a 58.2% increase. The low overall communication rates in East Hararghe reflect the more conservative gender norms prevailing among these communities.

Our qualitative findings also reflect these more restrictive norms, with more mixed changes in norms around menstruation. A significant number of girls emphasized that stigma around menstruation had reduced as a result of AWH-E programming. An 11-year-old girl from Community D explained: ‘*After we got information and knowledge about menstrual hygiene management there is nothing we are afraid of…we tell other friends that missing school because of this is foolishness'*. Several community health extension workers concurred, explaining that according to local cultural and religious norms, girls are expected to marry in very early adolescence, prior to menarche, but that AWH-E is helping to challenge this:

*Parents encourage girls to marry before they face the first menstruation period. I break [this norm] and teach that it is not haram [forbidden by the Qur'an]. They encourage a girl to marry at the age of 10 years but not at the age of 15 years so as to prevent her from facing her first menstruation period. They assume first menstruation means she is ready to bear a child …I told them it is not haram and menstruation can come anyways, and there is an age that a girl should marry and they accepted it … With the AWH-E programme, the awareness increased … There is a huge change*.

(Health extension worker, Community D).

However, a number of adolescent girls and female mentors underscored that this norm change process has also led to backlash in some communities and that discussions around adolescent puberty changes and sexual and reproductive health have prompted insults toward AWH-E programme participants from some community members. An 11-year-old girl from Community E noted that ‘*The mentor teaches us about menstruation, about relationships between boys and girls … When adolescent girls see us, they said, “are you crazy that you attend education of prostitute?” And they insult us saying “prostitute”*.' However, a 12-year-old girl from the same community explained that some girls highly valued the life skills education they were receiving and countered the negative reaction to the programme:

*Our peers say that, “You are attending the education of prostitutes”. They tell us it is very bad education. They don't want to come close to us because of that. But we tell them that we will attend it even if you call it prostitutes' education. We even attend if you try to kill us*.

(Girl, aged 12, Community E, AWH-E programme participant).

##### Changes in Knowledge About Menstruation and Biological Function

Unlike in South Gondar, AWH-E had significant quantitative impacts on knowledge among boys in East Hararghe (Table 4, Panel A). Specifically, boys in AWH-E communities were 11.6 percentage points (20.6%) more likely to know that menarche means a girl can get pregnant. It is worth noting that the control mean is substantially lower in East Hararghe (56.3 vs. 80.1%), thus providing more scope for impact. While not significant, treatment coefficients for boys are positive for the other two indicators.

For girls, we find large and statistically significant impacts for two of the three measures (Table 3, Panel A). Our results show that girls in AWH-E communities were 10.2 percentage points (29%, *p* < 0.001) more likely to know the frequency of menstruation and 9.0 percentage points (15.4%, *p* < 0.05) more likely to know that menarche allows for pregnancy. While control means are significantly lower for these two measures in East Hararghe compared to South Gondar, we see the opposite pattern for knowledge that girls reach puberty earlier than boys, with 72.5% of girls in control communities in East Hararghe correctly answering this question compared to 28.7% in South Gondar. This high level of baseline knowledge may partly explain the lack of treatment impact on this measure.

Our qualitative findings suggest that while there were improvements in girls' knowledge about MHM, broader knowledge gains on menstruation and the menstrual cycle were relatively limited in East Hararghe. Some adolescent girls noted that they were aware of the age at which they were likely to experience menarche, and that this was reinforcing information gained in biology class. A biology teacher from Community F further explained: ‘*I asked them what they had been learning in the [AWH-E] sessions and they told me they had been learning all the contents found in chapter three [of the biology textbook]. This means that they will not be shy when we start teaching about these issues in Grade 8. Often girls without prior knowledge bite their dresses [out of shame] when learning similar topics'*. A significant number also explained that they had learnt that with the right MHM practices, they did not need to stay away from school during menstruation.

*We have to prepare sanitary pads when we see our menstrual cycle. They – the AWH people – also told us that we have to wash and change the sanitary napkins every time … and that we do not have to miss school because of that …*

(Girl, aged 13, Community F).

That said, it is important to contextualize this knowledge and underscore that in the subset of communities in which the qualitative interviews were carried out, rates of girls' school attendance among very young adolescents were already very low, suggesting that the messaging may not be sufficiently aligned to girls' realities. One girl explained that:

*Now, since there are very few female students, no one misses school. For example, there are only two girls in grade 4. But in the past, they used to feel afraid and miss classes … I tell my friends that missing school because of seeing your period is foolishness … We do not know the age when we will get our period … They have not told us what age, but they instructed us how we should manage the cycle*.

(Girl, aged 12, Community F).

It is also notable that very few girls in the qualitative interviews appeared to have internalized accurate messaging from the curriculum about links between the menstrual cycle and pregnancy, and prevention approaches. For example, an 11-year-old girl from Community E noted that: ‘*We have become aware that we should take care of our reproductive organs, and not to swim in water where males swim when we are menstruating*.'

##### Changes in Knowledge and Behavior Related to Menstrual Hygiene Management

As noted earlier, our quantitative findings in this domain focused on behavior change related to MHM among girls who had already reached menarche. The sample size was small at baseline (112 girls), but results are promising (Table 3, Panel A). Across all three indicators, the coefficient is positive, and for the indicator for improved MHM, the impact is large and statistically significant. Girls in treatment communities are 26.2 percentage points (132%) more likely to report improved MHM than girls in the control group–a finding that is statistically significant at the 5% level. This finding highlights both the low levels of improved MHM in East Hararghe compared to South Gondar (in the control communities, 19.5 vs. 46.6%), and the strong role that AWH-E is playing in improving these measures.

Turning to the qualitative findings, compared to South Gondar, where adolescents' knowledge gains on MHM were often very detailed, qualitative interviews in East Hararghe suggested that improvements in knowledge were more general but nevertheless positive. Even though the vast majority of girls had not yet experienced menarche, those participating in AWH-E indicated that they had improved knowledge about MHM: ‘*To manage the menstruation flow with clean piece of cloth so that we can attend class conveniently. The menstruation flow will not spill down on our legs*' (12-year-old girl, Community F). Female mentors also noted that they themselves had gained knowledge about menstrual health and that this was one of the aspects of the programme they valued most:

*I learnt a lot of things about the development of girls. It has been very important to me personally. Even I didn't know how to handle menstruation before. Now I am knowledgeable and I can handle my menstruation effectively*.

(Female mentor, Community E).

Boys participating in the programme explained that through group discussions they too had gained important knowledge about how they could support girls during menstruation, and encourage them to continue attending school during their period. As a 13-year-old boy from Community F explained:

*We advise them [classmates not participating in AWH-E]. We are selected from each class and thus we advise them respectively … We advise them that they should not laugh at girls. We share with them all that we heard from the club… I also inform them that they should leave [go outside the classroom] if they see a girl menstruating … as it could make her shy and even force her to quit school*.

(Boy, aged 13, Community F).

##### Ability to Implement Improved Menstrual Health Management Knowledge in Context

While there was limited discussion among adolescent girls in East Hararghe about how they could apply what they had learned from the curriculum to their local realities, one message that appeared to resonate strongly among mothers and mentors was the link between improved MHM and girls' education. Mentors underscored that caregivers were now being more proactive in supporting girls during menstruation, as one female mentor explained:

*Initially, when the girls refused to go to school, their mothers seemed not to ask their reason for staying away from school. But now, as they have understanding about menstruation and its side effects, when the girls remain at home, their mother asks why she is not going to school and checks if that relates to menstruation or not*.

(Female mentor, Community D).

Whereas, improved knowledge among boys and their potential role as champions for girls in the context of MHM appeared less widespread than in the South Gondar sample, in Community F, boys explained that community leaders had worked with boys' group participants after the awareness sessions on girls' menstrual health to construct a menstrual hygiene facility at school in an effort to support girls' education.

*In the past, whenever female students experienced menstruation, there were no facilities available for them to access at school. After this project started … separate rooms are built so that female students can manage their menstrual hygiene. Now the school has a class where girls can rest, where they take a bath and where they discuss together their concerns … After the training, when we became aware about MHM, the kebele committee met and discussed how to put the training into practice on the ground. Consequently, we mobilised the community and … constructed the MHM rooms and the segregated latrine for the female students*.

(13-year-old boy, Community F).

## Discussion

The findings in this paper highlight the potential of programming aimed at supporting very young adolescents' knowledge and awareness about pubertal development and sexual and reproductive health in LMICs not only for improving menstrual health literacy among girl participants but also for shifting norms, knowledge and practices more widely. We argue that it is important to differentiate among different dimensions of menstrual health literacy as changes across these different dimensions may be impacted unevenly by programme interventions. These encompass changes to (1) stigmatizing norms about menstruation (which can contribute to a sense of shame and limited mobility among girls and women) (3, 26, 27); (2) knowledge of biological facts about menstruation (which are important, for example, in shaping knowledge and practices to prevent adolescent pregnancies) (24, 59); (3) knowledge about MHM (which is important in supporting girls' broader health and access to education) (47, 60); and (4) the ability to apply this new knowledge to local realities (including through efforts to provide local supplies and infrastructure that are supportive of MHM) (38, 51).

Our findings from these two zones in Ethiopia suggest that shifting norms around menstruation happened relatively quickly given synchronized programming with adolescent girls, boys and caregivers, and that there was good uptake of information about MHM practices. However, curriculum content about the biological facts of menstruation changed much more unevenly, and girls' ability to practice their new knowledge on MHM depended on the extent to which programme implementers and community leaders engaged directly with male students to construct separate latrines and changing facilities for girls. Where community leaders were actively engaged in the process, as in South Gondar, the impacts appeared transformational, but this required a high level of community buy-in, which was not always possible within the 2-year programme timeframe, and so may require a more sustained intervention. Indeed, as observed elsewhere (11, 12, 60), changes in norms, knowledge and behavior related to MHM can only be attained with the active involvement of all stakeholders.

Another key factor that emerged was the extent to which the programme was able to build on, reinforce and strengthen previous change efforts. Making connections between adolescent menstrual health and other health and gender issues is key to effective programming (68); this paper highlights the challenges of undertaking programming in contexts with different starting points when it comes to knowledge and behaviors. Recognition of how deprivations “cluster” (38, 51) is useful here: in East Hararghe, norms around menarche interact with poor infrastructure and low rates of schooling for girls to impact programming effectiveness. A critical difference between the two zones in this sample was the more widespread efforts in schools–both in biology classes and in girls' or gender clubs–in South Gondar, Amhara, to discuss issues related to puberty and menstruation, and upon which the AWH-E intervention was able to scaffold their more in-depth curriculum content and awareness-raising efforts. Moreover, messaging that focused on the links between good MHM and the continuity of girls' schooling had greater resonance in South Gondar because of higher average rates of school enrolment and of literacy (44.9%) among girls in upper primary school and beyond (69). This is in contrast to East Hararghe, where the school dropout rate for girls at this level was very high, and is also reflected in lower female literacy levels (37.3%) (69). The widespread misconception that menarche denotes sexual readiness, and thus that learning about menstruation is akin to learning about sex, presented a further challenge for girls who participated in the East Hararghe programme. These contextual differences–and their consequences for girls' behavior–underline the need for gender-transformative programming where content is adapted to address the specific socio-environmental conditions and norms that shape both girls' experiences of menstruation and their capabilities to apply new knowledge (3, 33, 45).

The paper had a number of important limitations. First, the majority of the girls in the sample had not reached menarche, limiting our ability to discuss impacts on MHM behavior. Second, these are short run findings, and future research is needed to assess whether these impacts are sustainable—both for the participants in the program as well as for broader normative change in these communities. Finally, East Hararghe and South Gondar are two unique settings, thus findings may not be externally valid in other contexts.

With these limitations in mind, the findings make two important contributions to the literature. First, they provide novel empirical evidence about menstrual health literacy among very young adolescents–a cohort that tends to be overlooked by large-scale national surveys that focus on girls and women aged 15 and older (46). Second, the impact evaluation evidence contributes to our understanding of the pathways through which integrated life-skills programming targeting very young adolescents can bring about shifts in norms, knowledge and practices related to menstrual health and wellbeing, not only among girls but also their male peers, caregivers, mentors and the wider community in low-income settings within a relatively short time horizon– suggesting a promising opportunity for broader transformation of the gender norms that restrict girls' capabilities as they transition from childhood to adulthood (49). Given the important health, education and empowerment spillover effects of menstrual health literacy (5, 14, 15, 48, 56, 68), this evidence suggests that interventions in this area could accelerate wider development gains for girls, women and their communities.

## Data Availability Statement

The raw data supporting the conclusions of this article will be made available by the authors, without undue reservation.

## Ethics Statement

The studies involving human participants were reviewed and approved by George Washington University Committee on Human Research's Institutional Review Board (071721); the Overseas Development Institute Research Ethics Committee (02438); the Ethiopian Development Research Institute (EDRI/DP/00689/10); the Addis Ababa University College of Health Sciences Institutional Review Board (113/17/Ext). Written informed consent for participation was not provided by the participants' legal guardians/next of kin because we have obtained written or verbal consent depending on the literacy level of the respondent.

## Author Contributions

Study conception and design: SB, JH, NJ, and TW. Data collection: KG and WY. Analysis and interpretation of results: SB, JH, KG, WY, and NJ. Draft manuscript preparation: SB, JH, KG, KP, WY, TW, and NJ. All authors reviewed the results and approved the final version of the manuscript.

## Funding

The research underpinning this article was funded through the Gender and Adolescence: Global Evidence (GAGE) research consortium by UK aid.

## Conflict of Interest

WY was employed by the company Quest Consulting. The remaining authors declare that the research was conducted in the absence of any commercial or financial relationships that could be construed as a potential conflictof interest.

## Publisher's Note

All claims expressed in this article are solely those of the authors and do not necessarily represent those of their affiliated organizations, or those of the publisher, the editors and the reviewers. Any product that may be evaluated in this article, or claim that may be made by its manufacturer, is not guaranteed or endorsed by the publisher.

## Footnotes

^1^Note that studies of school-based random samples of girls elsewhere in rural Ethiopia suggest an average age of menarche of 13.9-14.1 years (24, 39).

^2^The two additional study treatment arms, which we do not consider in the present analysis, are: (1) a different curriculum-based safe spaces programme for girls only; and (2) AWH-E programming combined with asset transfers for girls. Communities in (1) are excluded in order to focus the analysis on impacts of a multi-level program that aimed to transform the lives of girls through a more holistic community-oriented approach – by interacting with girls as well as their social networks (male peers, parents, and broader communities). Comparison of this holistic approach and a simpler program that intervenes with adolescent girls only is left for future work. Similarly, communities in (2) are excluded from the present analysis in order to focus on impacts of multi-level, gender transformative curriculum-based programming itself, without study of the possible additional impacts of asset transfers to girls.

^3^Note that there is some variation in sample sizes across outcomes in the main analysis tables. This is primarily due to the sample of individuals who should have responded to each question; for instance, the indicator for adolescent has ever talked to mother/female guardian about menstruation was only asked among those girls living with a female guardian at the time of data collection, and the MHM questions were only asked among girls who had reached menarche (13% of our sample). There are also very minor differences across outcomes due to missing data (adolescents who refused to respond to a particular question, which was extremely rare in our data.

^4^The asset index takes a value between 1 and 9 indicating the decile of household assets in relation to the rest of the GAGE sample in Ethiopia (based on a series of indicators for 16 different measures of household durables). The Productive Safety Net programme is a governmental assistance program that offers public works employment and/or food aid to poor and chronically food insecure households–we use this measure as a proxy for household vulnerability.
